# The First High-Density Genetic Map Construction in Tree Peony (*Paeonia* Sect. *Moutan*) using Genotyping by Specific-Locus Amplified Fragment Sequencing

**DOI:** 10.1371/journal.pone.0128584

**Published:** 2015-05-26

**Authors:** Changfu Cai, Fang-Yun Cheng, Jing Wu, Yuan Zhong, Gaixiu Liu

**Affiliations:** 1 Landscape Architecture College of Beijing Forestry University, National Flower Engineering Research Centre, Beijing, China; 2 National Peony Garden, Luoyang, Henan, China; Pennsylvania State University, UNITED STATES

## Abstract

Genetic linkage maps, permitting the elucidation of genome structure, are one of most powerful genomic tools to accelerate marker-assisted breeding. However, due to a lack of sufficient user-friendly molecular markers, no genetic linkage map has been developed for tree peonies (*Paeonia* Sect. *Moutan*), a group of important horticultural plants worldwide. Specific-locus amplified fragment sequencing (SLAF-seq) is a recent molecular marker development technology that enable the large-scale discovery and genotyping of sequence-based marker in genome-wide. In this study, we performed SLAF sequencing of an F1 population, derived from the cross *P*. *ostti* ‘FenDanBai’ × *P*. × *suffruticosa* ‘HongQiao’, to identify sufficient high-quality markers for the construction of high-density genetic linkage map in tree peonies. After SLAF sequencing, a total of 78 Gb sequencing data and 285,403,225 pair-end reads were generated. We detected 309,198 high-quality SLAFs from these data, of which 85,124 (27.5%) were polymorphic. Subsequently, 3518 of the polymorphic markers, which were successfully encoded in to Mendelian segregation types, and were in conformity with the criteria of high-quality markers, were defined as effective markers and used for genetic linkage mapping. Finally, we constructed an integrated genetic map, which comprised 1189 markers on the five linkage groups, and spanned 920.699 centiMorgans (cM) with an average inter-marker distance of 0.774 cM. There were 1115 ‘SNP-only’ markers, 18 ‘InDel-only’ markers, and 56 ‘SNP&InDel’ markers on the map. Among these markers, 450 (37.85%) showed significant segregation distortion (P < 0.05). In conclusion, this investigation reported the first large-scale marker development and high-density linkage map construction for tree peony. The results of this study will serve as a solid foundation not only for marker-assisted breeding, but also for genome sequence assembly for tree peony.

## Introduction

Belonging to the Paeoniaceae, tree peonies (*Paeonia* Sect. *Moutan*), native to China, are believed to have been cultivated as ornamental and medicinal plants for over 1600 years [1, 2]. Due to their varying forms of flowers, rich palette of horticultural varieties, and deep ethnobotanical history in Chinese culture, tree peonies have been referred to as ‘the king of flowers’, and have now been widely cultivated as important horticultural crops in many countries of Asia, America, Europe, and Australia [1–3]. To date, approximately 2100 cultivars of tree peony have been grown throughout the world, and more than 1000 cultivars are found in China [1, 4–6]. In addition, tree peony seeds have been recently identified as a novel resource of high-quality edible oil with rich unsaturated fatty acids, such as α-linolenic acid, oleic acid, and linoleic acid [7, 8]. Moreover, their complicated genetic structure, wide geographic distribution, long cultivation history, and abundant genetic variation, has made them a useful evolutionary model [9–11].

As a group of important species for horticultural cultivation and biological research, tree peonies have received more and more attention in recent years. The studies of genetic diversity and relatedness [12–14], cultivar identification [15, 16], and hybrid origin [9] in tree peonies, had been done using molecular markers. Despite these progresses in genetic researches of tree peonies, the genetic and molecular mechanisms of their ornamental and biological traits were still poorly understood. Genetic linkage maps have become significant tools for elucidating the genome structure and identifying molecular markers linked to traits [17]. The construction of genetic linkage maps has been performed in many ornamental plants [18–22]. As for tree peonies, no genetic map has been developed until now. Lack of sufficient user-friendly molecular markers is one of the major causes hindering the development of genetic map in tree peonies.

Among molecular markers, single nucleotide polymorphisms (SNPs) are the most useful molecular markers because they are the most abundant and frequent type of genetic variation in genomes [17, 23]. The development of next generation sequencing (NGS) make it possible to rapidly identify a large number of SNPs in the genome. In the beginning, whole-genome re-sequencing was used for SNP identification and genetic mapping in a few organisms that have a relatively small genome size [24], but it is not effective for the majority of organisms with a large genome and no reference genome sequence. Consequently, using restriction-site associated DNA (RAD) sequencing, Miller et al. [25] developed a cost-effective method for SNP detection and high-throughput genotyping. This method has been used for SNP identification and genetic mapping in a quantity of plant species, including grape [26], *Lolium perenne* [27], and barley [28]. Subsequently, many modifications of RAD sequencing to make it more effective and economical have been reported [29–31]. Recently, specific-locus amplified fragment sequencing (SLAF-seq), a high-resolution strategy of large-scale *de novo* SNP discovery and genotyping, was first described by Sun et al. [32]. The strong power of SLAF-seq for genetic research has subsequently used in the development of SLAF markers in *Thinopyrum elongatum* [33] and maize [34], and for the construction of high-density genetic maps in sesame [35] and soybean [36]. Therefore, it is clear that SLAF-seq is the optimal choice for large-scale molecular marker development and high-density linkage map construction, especially in organisms for which no reference genome information is available.

We performed a tree peonies hybridization breeding research seven years ago, which established a large number of segregation populations for tree peonies. After a four years field investigation, we selected an optimizing segregation population, derived from the cross *P*. *ostti* ‘FenDanBai’ × *P*. × *suffruticosa* ‘HongQiao’, for genetic linkage mapping. The main aim of this investigation was to discover large-scale genome-wide molecular markers and construct a high-density linkage map in tree peony. We exploited SLAF-seq approach to identify large-scale molecular markers and generate genotype data from this segregation population. Subsequently, using these data, we constructed the first high-density linkage map for tree peony. In addition, characteristics of these SLAF markers and linkage map were investigated. The availability of such large numbers of SNP markers and the high-density linkage map will serve as a solid foundation not only for marker-assisted selection (MAS) breeding, but also for positioning scaffolds arising from whole-genome sequencing projects in tree peony.

## Materials and Methods

### Plant materials and DNA extraction

An F_1_ mapping population of 195 individuals, derived from a cross of *P*. *ostti* ‘FenDanBai’ (female parent) and *P*. × *suffruticosa* ‘HongQiao’ (male parent), was used to construct the genetic linkage map. The parents were advanced selected from Luoyang National Peony Garden, Luoyan, China (34°43'N, 112°24'E), and differ in many growth and morphological traits. The F_1_ mapping population plants were grown in the Beijing Guose Peony Garden, Beijing, China (40°28'N, 116°4'E).

Total DNA was extracted from young leaves of each individual, stored in plastic bags with silica gel, and kept at room temperature until analysis, according to the plant genomic DNA extraction kit (TIANGEN, Beijing, China) and following the manufacturer’s instructions. DNA quality was estimated by electrophoresis on 1% agarose gels with a lambda DNA standard. DNA concentration, which should be greater than 50 ng/μl, was measured with the ND-1000 spectrophotometer (NanoDrop, Wilmington, DE, USA). A minimum of 2 μg of DNA were used for specific-locus amplified fragment sequencing (SLAF-seq).

### SLAF library construction and high-throughput sequencing

A total of 197 individuals, including the two parents and 195 progeny, were *de novo* genotyped using SLAF sequencing, as described by Sun et al. [32], with a few modifications. In brief, (1) genomic DNA from each sample was incubated with *Mse*I (NEB, Ipswich, MA, USA), T4 DNA ligase (NEB), ATP (NEB), and *Mse*I adapter at 37°C. Restriction-ligation reactions were heat-inactivated at 65°C, and then digested with the additional restriction enzyme *Nla*III at 37°C. (2) These restriction-ligation reactions were diluted in 30 μl elution buffer and mixed with dNTPs, Taq DNA polymerase (NEB), and *Mse*I-primer containing barcode 1 for polymerase chain reaction (PCR). The PCR products were purified by using E.Z.N.A. Cycle Pure Kit (Omega, UK). (3) The purified PCR products were pooled and incubated at 37°C with *Mse*I, T4 DNA ligase, ATP, and Solexa adapter. After incubation, the reaction products were purified using a Quick Spin column (Qiagen, Venlo, Netherlands), and electrophoresed on a 2% agarose gel. (4) Fragments of 380–430 bp (with indexes and adaptors) in size were isolated using a gel extraction kit (Qiagen). These fragments were then subjected to PCR with Phusion Master Mix (NEB) and Solexa Amplification primer mix (Illumina, Inc., San Diego, CA, USA) to add barcode 2, following the Illumina sample preparation guide. (5) PCR products(SLAFs) of 380–430 bp, were gel purified and diluted in 30 μl elution buffer for pair-end sequencing on an Illumina High-seq 2500 sequencing platform (Illumina, San Diego, CA, USA), at Biomarker Technologies Corporation in Beijing (http://www.biomarker.com.cn/). (6) In order to control the quality of the sequencing data, real-time monitoring was carried out for each cycle during Illumina sequencing, and two key indicators were calculated, including the ratio of high quality bases with quality scores greater than Q20 (i.e. a quality score of 20, indicating a 1% chance of an error and, thus, 99% confidence) in the raw reads and guanine–cytosine (GC) content.

### SLAF-seq data grouping and genotype definition

Analysis of SLAF-seq data was mainly divided into two parts, reads clustering and alleles definition. All SLAF pair-end reads with clear index information were clustered together, based on sequence similarity which was detected using one-to-one alignment by BLAT (-tileSize = 10—stepSize = 5) [37]. Sequences with over 90% identity were defined as a SLAF locus [32]. In each of the SLAF, we found alleles between the parents using the minor allele frequency (MAF) evaluation by the software developed by Sun et al. [32]. There are three types of marker in SLAF loci, including SNPs (SNP-only), insertion–deletion (InDel-only), and SNP&InDel. All polymorphism SLAF loci were genotyped with consistency in the offspring and parental.

All SLAF marks had been filtered and quality assessed many times by the method described by Sun et al. [32]. (1) SLAFs containing more than four tags were filtered out as repetitive SLAFs, because tree peony is a diploid species, one locus contains at most four SLAF tags. (2) SLAFs that had less than three SNPs, and average depths of each sample above three, were considered as high quality SLAFs. (3) These high quality SLAFs with two, three, or four tags were identified as polymorphic SLAFs and considered to be potential markers. (4) Polymorphic SLAF markers were classified into eight segregation patterns (*aa × bb*, *ab × cc*, *ab × cd*, *cc × ab*, *ef × eg*, *hk × hk*, *lm × ll*, and *nn × np*). Because the F_1_ population of tree peony is considered as a cross-pollinator (CP) population [38], only the SLAF markers, whose segregation patterns were *ab × cd*, *ef × eg*, *hk × hk*, *lm × ll*, *nn × np*, *ab × cc*, and *cc×ab*, were used for high-density genetic map construction. (5) In a final filtering step, SLAF markers with average sequence depths of more than 20-fold in parents and more than 3-fold in progeny, and with integrity of more than 70% in mapping population individuals, were selected for use in genetic mapping.

### Genetic linkage map construction

For linkage analysis, the sequencing data of the successful SLAF makers were utilized. The chi-square test (χ^2^) was performed to test deviation of polymorphic markers from Mendelian inheritance ratios (*P* < 0.05). Markers showing segregation distortion were also integrated into the map, and the regions with more than three adjacent loci revealing skewed segregation (*P* < 0.05) were identified as segregation distortion regions (SDR) [39]. Considering that data of next generation sequencing (NGS) may have many genotyping errors and deletion, which can greatly affect the linkage maps quality, HighMap strategy was employed to order SLAF markers and correct genotyping errors [40]. Markers were divided into linkage groups (LGs), using the single-linkage clustering algorithm at logarithm of odds (LOD) threshold ≥4.0 and a maximum recombination fraction of 0.4. To order markers correctly, the processes of marker ordering and error genotype correction were carried out iteratively. As four or more cycles, high-quality maps were constructed. Map distances were estimated for each LG using the Kosambi’s mapping function [41], and denoted in centiMorgans (cM).

## Results

### Analysis of SLAF sequencing data

High-throughput sequencing of the SLAF library generated 78 Gb of data containing 285,403,225 pair-end reads (S1 Table). After eliminating the index sequences, both ends of each read was about 30 bp length. On average, 74.96% of these sequencing bases were high-quality, with quality scores greater than Q20. GC content of 197 sequencing samples raged from 42.34% to 43.50% with an average of 42.89%. To enhance the chances of detecting segregating markers in the parents, the parents were sequenced at a substantially higher level than their progeny. Therefore, the number of reads for male and female parent was 9,176,662 and 11,131,195, respectively. Whereas, the number of reads for the 195 progeny ranged from 709,524 to 3,011,586 with an average of only 1,359,463 (S1 Table).

### SLAF markers detection and genotype definition

**Fig 1:**
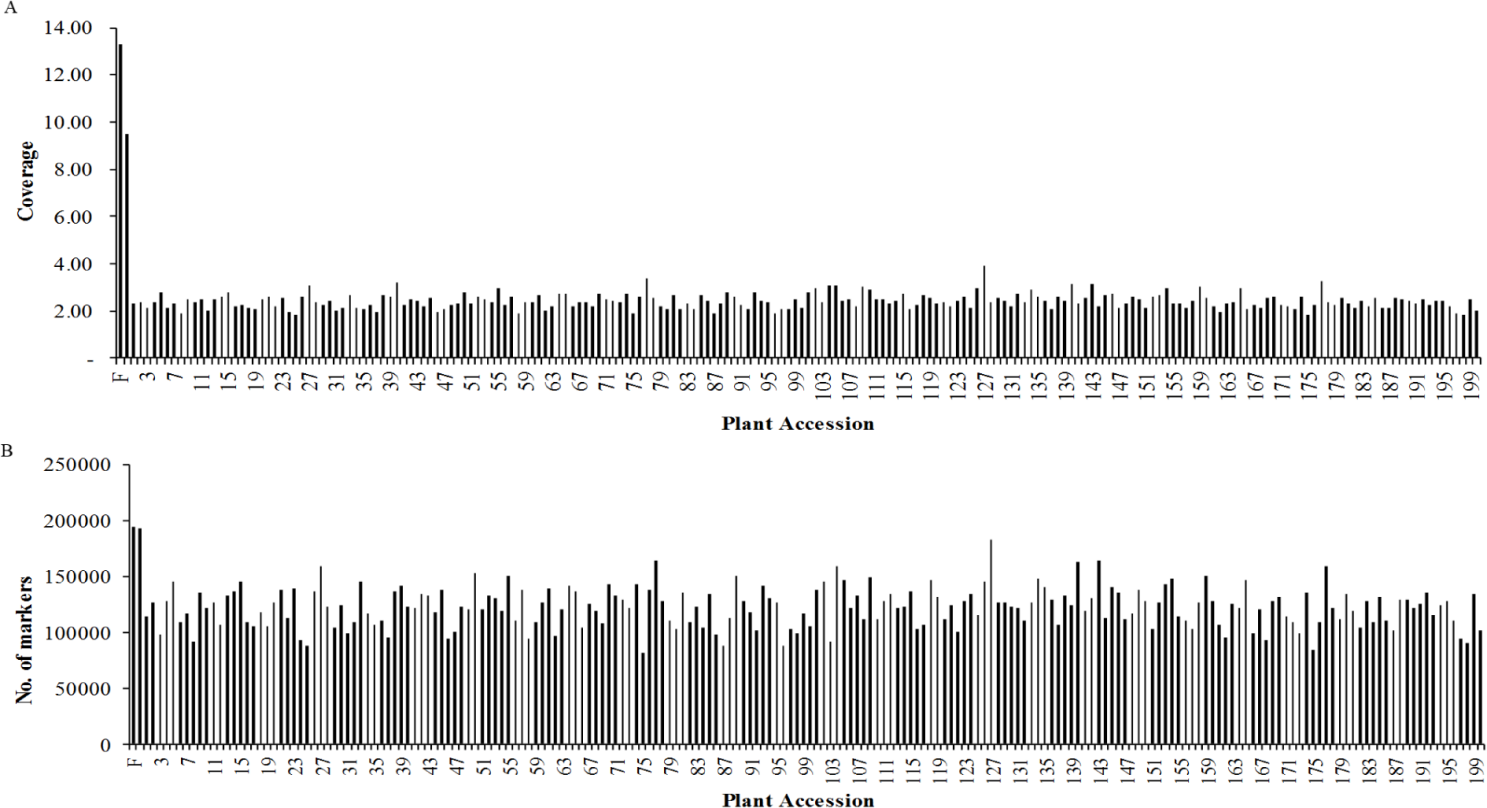
Coverage and number of markers for each of F_1_ progeny individual and two parents. The *x*-axes in both **A** and **B** indicate the plant accession including the female parent and the male parent followed by 195 F_**1**_ progeny individuals, the y-axes indicates coverage in **A** and number of markers in **B**.

Based on sequence similarity, all reads were clustered into SLAFs. Excluded the low-depth and repeat-suspicious SLAFs, a total of 309,198 high-quality SLAFs were defined with 64,633,032 reads. Of these high-quality SLAFs, 192,879 were detected in the male parent, and 194,377 were detected in the female parent, and the average coverage for each SLAF from the parents was 9.5-fold and 13.28-fold, respectively. In the 195 progeny of the mapping population, the number of SLAFs in each progeny ranged from 82,488 to 183,358 with an average of 123,051, and the coverage of SLAFs in each progeny ranged from 1.82-fold to 3.91-fold with an average of 2.41-fold (Fig 1).

**Fig 2:**
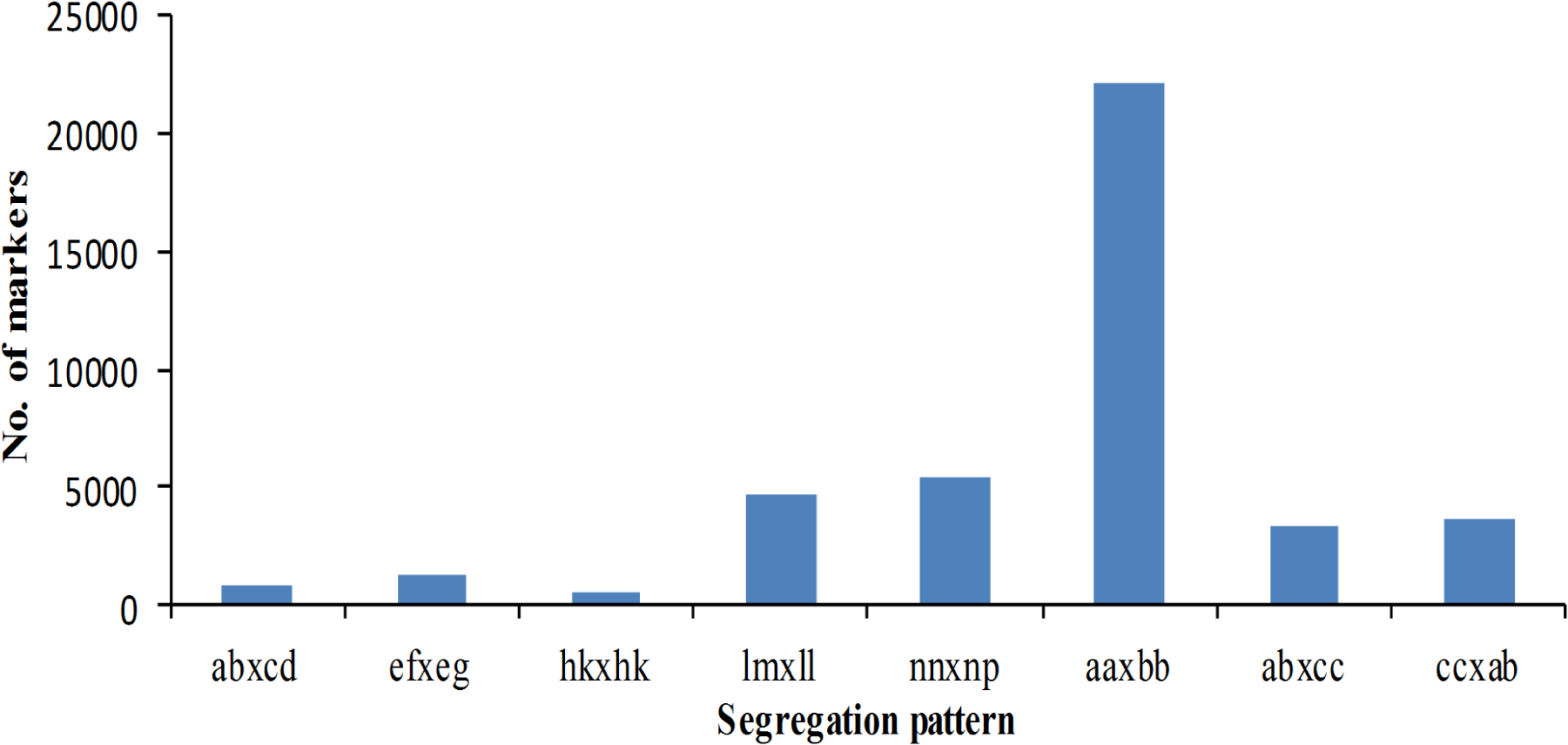
Distribution of SLAF markers in eight segregation patterns.

Among the 309,198 high-quality SLAFs that were defined, 85,124 were polymorphic, while other 224,074 were non-polymorphism. The polymorphism rate of these high-quality SLAFs was only 27.5% (Table 1). After filtering out the SLAFs lacking parent information, 42,085 of these polymorphic SLAFs were obtained and successfully classified into eight segregation patterns (Fig 2). As show in Fig 2, over 50% of markers were homozygous in two parents with genotype *aa* or *bb*, which were unsegregated in the progeny. Only 19,966 makers (47.44%) conformed to the CP population segregation codes, including *ab × cd*, *ef × eg*, *hk × hk*, *lm × ll*, *nn × np*, *ab × cc*, and *cc × ab*. After filtered low quality SLAF markers, which average sequence depths were less than 20-fold in parents and less than 3-fold in progeny, and integrities less than 70% in individuals, 3518 of these 19,966 markers were defined as effective markers and used for genetic linkage mapping. The segregation types for these effective markers were shown in Table 2. Of these 3518 markers, 1608 markers (45.71%) were homozygous in female parent and heterozygous in male parent, 1735 markers (49.32%) were homozygous in male parent and heterozygous in female parent, and only 175 markers (4.97%) were heterozygous in both parents. Average sequencing depths of these 3518 markers were 29.85-fold in the female parent, 20.36-fold in the male parent, and 3.12-fold in each progeny (Table 3).

**Table 1:** Initial detection of SLAF markers.

	Number of SLAF markers	Number of reads	Ratio
**Polymorphism**	85,124	17,748,865	27.5%
**Non-polymorphism**	224,074	46,884,167	72.5%
**Total**	309,198	64,633,032	100%

**Table 2:** Statistic of the segregation types for SLAF markers.

Type	Marker number	Percentage (%)
**ab × cd**	12	0.34
**ef × eg**	121	3.44
**hk × hk**	42	1.19
**lm × ll**	998	28.37
**nn × np**	965	27.43
**ab × cc**	737	20.95
**cc × ab**	643	18.28
**Total**	3518	100.00

**Table 3:** Summary of effective SLAF markers depths.

Samples	SLAF marker number	Total depth	Average depth
**Female parent**	3518	105005	29.85
**Male parent**	3518	71611	20.36
**Average of Offispring**	3518	10965	3.12

### Construction of high-density linkage map

A total of 3518 demonstrably heterozygous SLAF markers were available for mapping. After linkage analysis, 1189 of these SLAF markers (S2 Table) were mapped onto five linkage groups. Therefore, the mapping ratio of these SLAF markers was about 33.80%. Based on an analysis on the 1189 SLAF markers, we found that the average sequencing depths of these SLAF markers were 32.01-fold in the male parent, 48.18-fold in the female parent, and 4.87-fold in each offspring. In addition, the integrity of these markers among the 195 F_1_ individuals was 78% on average, which was an important indicator for controlling the quality of linkage map.

Finally, we constructed a high-density, integrated linkage map of the tree peony, comprising 1189 markers distributed over five linkage groups (Table 4, S1 Fig). The map spanned 920.699 cM with an average inter-marker distance of 0.774 cM. The genetic length of LGs ranged from 108.947 cM (LG3) to 296.431 cM (LG2), with an average of 184.140 cM. LG2 was the most saturated, having 368 markers with an average marker density of 0.806 cM, whereas LG4 had the least number of markers (only 153). Moreover, ‘Gap ≤5’ (that is, indicated the percentages of gaps where the distance between adjacent markers was smaller than 5 cM.) of each linkage group ranged from 97.83% to 98.88% with an average of 98.49%, which reflected the degree of linkage between markers. In total, there were 13 gaps that were 5 to 10 cM in length and six gaps larger than 10 cM. The largest gap was located in LG1 with 27.409 cM in length (Table 4).

**Table 4:** Summary of integrated linkage map of tree peony.

Linkage group	Number of marker				Size (cM)	Average distance (cM)	Gaps≤5	Maximum gaps (cM)
	SNP-only	InDel-only	SNP&InDel	Total				
**LG1**	231	3	12	246	197.22	0.802	98.37	27.409
**LG2**	348	8	12	368	296.431	0.806	97.83	10.013
**LG3**	139	1	14	154	108.947	0.707	98.70	6.191
**LG4**	145	2	6	153	120.271	0.786	98.69	6.611
**LG5**	252	4	12	268	197.83	0.738	98.88	16.557
**Max LG**	348	8	12	368	296.431	0.806	97.83	10.013
**Min LG**	139	1	14	154	108.947	0.707	98.70	6.191
**Total**	1,115	18	56	1,189	920.699	0.774	—	—
**Average**	223	3.6	11.2	237.8	184.140	—	98.49	—

‘Gaps≤5’ indicated the percentages of gaps which the distance between adjacent markers was smaller than 5 cM.

### Distribution of markers types on the genetic map

**Fig 3:**
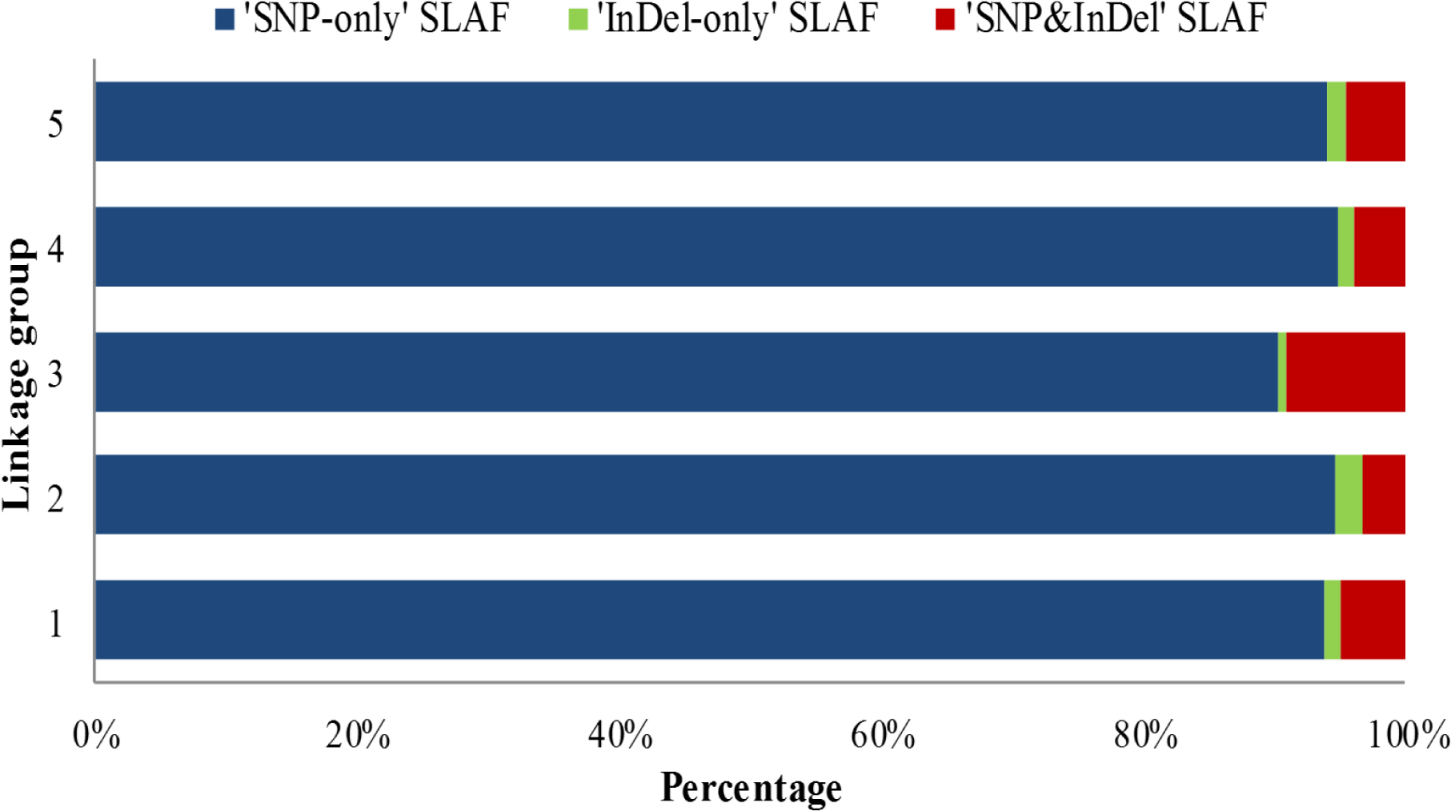
Percentages of diverse SLAF types on each linkage group.

The integrated genetic map of the tree peony had 1189 markers, including 1115 ‘SNP-only’ SLAF, 18 ‘InDel-only’ SLAF, and 56 ‘SNP&InDel’ SLAF, with percentages of 93.78%, 1.51%, and 3.87%, respectively. All categories of markers were found on each of LGs (Table 4, Fig 3). For instance, the percentages of the three types of markers on LG2, the largest linkage group, were 94.57%, 2.17%, and 3.26%, respectively. Among the five LGs, LG3 had the lowest percentage of ‘InDel-only’ SLAF at 0.65%, but had the highest percentage of ‘SNP&InDel’ SLAF, at 9.09%. ‘SNP-only’ SLAF markers were the most common of the three marker types in all LGs comprising between 90.85% and 94.57%.

The SLAF markers of ‘SNP-only’ and ‘SNP&InDel’ type were investigated further. The results showed that, among the 1115 SLAF markers of the ‘SNP-only’ type, 656 had more than one SNP loci. In total, 2518 SNP loci were detected among the 1115 ‘SNP-only’ SLAF and 56 ‘SNP&InDel’ SLAF on the final map. In addition, the percentages of different SNP types of these 2518 SNP loci were investigated (Table 5). Transition type SNPs were in the majority, accounting for 67.72%, including R (A/G) and Y (C/T) with percentages of 33.76% and 33.96%, respectively. Whereas, transversion type SNPs were in the minority, accounting for only 32.29%, including M (A/C), W (A/T), S (C/G), and K (G/T) with percentages ranging from 4.25% to 11.38%.

**Table 5:** Statistic of six types of mapped SNPs.

type	number	ratio
**M(A/C)**	287	11.38%
**W(A/T)**	164	6.51%
**S(C/G)**	107	4.25%
**R(A/G)**	850	33.76%
**Y(C/T)**	855	33.96%
**K(G/T)**	255	10.13%
**Total**	2518	100%

### Segregation distortion markers on the map

The results of χ^2^ test indicated that 450 (37.85%) of the 1189 markers showed significant segregation distortion (*P* < 0.05) on the integrated linkage map (S1 Fig). Furthermore, the distorted markers were found to be widely distributed on each linkage group, even though the ratios varied from one LG to another (Table 6). The frequency of segregation distortion markers on LG4 and LG5 was much higher than other LGs at 51.63% and 53.36%, respectively. LG2, the largest LG with 368 markers and 296.431 cM, had 125 distorted markers (33.97%). LG3 had 58 segregation distortion markers (37.66%). The lowest frequency of segregation distortion markers (18.29%) was LG1.

**Table 6:** Distribution of segregation distortion markers on the five linkage groups.

Linkage group	All marker number	All marker percentage	Segregation distortion marker number	Segregation distortion marker percentage	Frequency of segregation distortion marker	SDR number
**1**	246	20.69%	45	10.00%	18.29%	6
**2**	368	30.95%	125	27.78%	33.97%	15
**3**	154	12.95%	58	12.89%	37.66%	14
**4**	153	12.87%	79	17.56%	51.63%	7
**5**	268	22.54%	143	31.78%	53.36%	12
**Total**	1189	100%	450	100%	37.85%	54

‘SDR’ indicated segregation distortion regions

Of 450 distorted markers, 421 showed clustered distribution in 54 SDRs, of which six were located on LG1, 15 on LG2, 14 on LG3, seven on LG4, and 12 on LG5 (Table 6, S1 Fig). Of these, eight large SDRs, which clustered more than 10 distorted markers, were distributed on each LG, excluding LG1. The largest SDR, clustering 33 distorted markers, was located on LG5.

**Fig 4:**
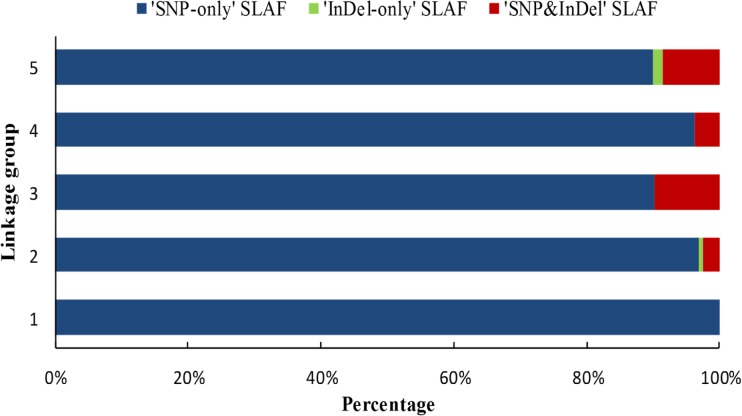
Percentages of diverse segregation distortion SLAF types on each linkage group.

The 450 segregation distortion markers contained all categories of markers, including 423 ‘SNP-only’ SLAF, three ‘InDel-only’ SLAF, and 24 ‘SNP&InDel’ SLAF, with percentages of 94.00%, 0.67%, and 5.53%, respectively. The percentages of three categories markers in these 450 segregation distortion markers were closed to the percentages observed for 1189 markers of three categories. However, three categories of segregation distortion markers were distributed differently for each LG (Fig 4). For example, only one category of segregation distortion markers, ‘SNP-only’ SLAF, was observed on LG1, whereas, all three categories of segregation distortion markers were distributed on LG2 and LG5.

## Discussion

### SLAF sequencing and the development of markers

Here, we used the SLAF sequencing approach to identify a set of SLAF markers in tree peony. We constructed a SLAF library of tree peonies, and obtained 78 Gb data containing 285,403,225 pair-end reads from this library. Subsequence, 309,198 SLAF markers were detected, and 3518 polymorphic markers were identified as effective markers for linkage mapping. The availability of a large number of molecular markers is essential for genetic research, especially for constructing a useful high-density linkage map. Traditionally, Amplified fragment length polymorphism (AFLP) and microsatellite markers were priority selections for linkage analyses in organisms lacking enough genomic information. He et al. [19] constructed the first genetic linkage map of crape myrtle (*Lagerstroemia*) by using AFLP and SSR. Yu et al. [42] constructed a genetic linkage map in tetraploid roses by the same way. However, these traditional markers are inefficient, expensive, and time-consuming for construction of high-density linkage maps which need thousands of markers [43]. In contrast to these traditional approaches of developing markers, the SLAF sequencing provided a rapid, accurate, economical and effective method for developing molecular markers [32]. Therefore, SLAF sequencing method has been successfully used in many plants for molecular markers development and genetic map construction, including soybean [36], cucumber [44], and sesame [35]. The results of this study further showed that SLAF sequencing method can provide useful genomic resources for large-scale molecular markers development and high-density linkage map construction. Moreover, these newly-developed markers will be severed as a useful molecular tool for other genetic studies, such as genetic diversity studies, genetic relationship analysis, and germplasm identification [45].

One advantage of the SLAF-seq method is that it can detect a large-scale markers in a single experiment. However, due to some unavoidable erroneous and missing values were contained in SLAF sequencing data, molecular markers developed in this approach must be stringently filtered to avoid false positive markers [32, 35]. In this study, 85,124 polymorphic SLAFs were discovered initially in the sequence dataset, but only 3518 SLAFs considered as effective markers after filtering out the SLAFs with missing genotypes, low integrity, Mendelian errors, or significant segregation distortion. In addition, we found that the accuracy of SLAF markers were improved with the increasing of sequence coverage. This result was similar to previous studies [32, 46]. Therefore, in order to improve the efficiency of marker development in future experiments, improving sequence coverage is necessary.

We analysed the sequencing dataset and found that GC content of these data was 42.23% on average, a little lower than the results showed in many sequenced transcriptomes of tree peonies [45, 47], possibly due to the different source of DNA sequences used (i.e. genome DNA, cDNA, or EST). We investigated the types of SNP loci of SLAF markers, and found the majority to be transition type SNPs (67.72%). This is similar to that observed in *Sesamum indicum* [35]. The polymorphism rate of SLAF markers between the two parents of this mapping population was 27.5%, lower than the polymorphism rate of the EST-SSR (39.9%) [48]. However, the polymorphism rate of SLAFs in tree peonies was higher than that reported for many other species [32, 34, 40, 48], indicating that the genetic diversity between germplasm resources of the tree peonies is high, similar to the previous study [11]. Thus, the results of SLAF sequencing generated a rich of genomic information for tree peonies, which accurately reflect the characteristics of genomic and genetic diversity of this species. This study further proves the utility of SLAF-seq in the genomic research of an organism without a reference genome sequence.

### Construction and characteristics of the genetic linkage map

Construction of genetic linkage map is more complicated in heterozygous perennial woody plants than in homozygous annual herbaceous plants [49]. For woody plants, due to their high heterozygosity, self-incompatibility, long-generation interval, it is impossible to build a conventional segregating progeny derived from two homozygous inbred lines [50]. So, genetic linkage maps of woody plants are generally constructed using an F_1_ full-sibling family, which has more types of segregating markers than conventional segregating progeny. In this study, an F_1_ full-sibling family, derived from the cross *P*. *ostti* ‘FenDanBai’ × *P*. × *suffruticosa* ‘HongQiao’, was used for the tree peony map construction. In addition, the tree peony is believed to have a very large genome, approximately 16G [51], which also complicated the construction of the high-density genetic map [52]. Therefore, use of suitable algorithms for constructing high-density linkage mapping is essential.

Here, we employed the HighMap method, which uses an iterative ordering and error correction strategy based on a k-nearest neighbour algorithm and a Monte Carlo multipoint maximum likelihood algorithm [40], for constructing a high-density linkage map in tree peony. Compared to the traditional mapping software JoinMap 4.1, HighMap permitted the utilization of more NGS data, constructed the linkage map of higher marker order accuracy and map distance stability, and had the ability of higher computational efficiency of map construction [40]. This method was successfully employed in common carp [32], and soybean [36]. The results of this study also showed that HighMap was a powerful tool for high-density linkage map construction. Compared the mapping results of HighMap and JoinMap4.1, we found that the linkage map of tree peony generated by HighMap had more markers and smaller map distance than that JoinMap4.1 created, which was 1189 markers, 920.699cM, and 1091 markers, 3868.094cM, respectively (Table 7, S3 Table). Furthermore, there are some minor difference of marker order between the linkage maps constructed by HighMap and JoinMap4.1 (S3 Table).

**Table 7:** Genetic linkage map of tree peony constructed by HighMap and JoinMap4.1.

Linkage groups	HighMap		JoinMap4.1	
	Marker numbers	Distances (cM)	Marker numbers	Distances (cM)
**LG1**	246	197.22	229	818.680
**LG2**	368	296.431	338	1200.507
**LG3**	154	108.947	142	595.148
**LG4**	153	120.271	144	517.861
**LG5**	268	197.83	238	735.898
**Total**	1189	920.699	1091	3868.094

We present here the first high-density linkage map of tree peony, contained 1189 SLAF markers. The linkage map contained five linkage groups, congruent with the karyotypes of *P*. *ostti* and *P*. × *suffruticosa* (2*n* = 10) [2]. The map spans 920.699 cM with an average distance of 0.774 cM between adjacent markers, with an average number of 237.8 markers per LG. The number of SLAF markers on each LG was different. And many markers were highly clustered on some regions of the map, especially on LG1, LG2, and LG5. This phenomenon may due to the non-random distribution of markers and the uneven marker polymorphism and recombination rates between mapping parents on some chromosomes [20]. The similar results were also reported in sunflower [53], grape [54], and tomato [55]. In addition, Ma et al. [55] considered that marker clusters were generally associated with the chromosome pericentromeric or heterochromatin regions. Moreover, despite the average distance between adjacent markers on the map were short (only 0.774 cM), there were six gaps larger than 10 cM, of which four were located in LG1, one on LG2, and one on LG5. These large gaps may be due to the lack of marker polymorphism and a shortage of markers detection in these regions [20, 56, 57].

Segregation distortion is a common phenomenon in many organisms [58], and is recognized as a potentially powerful evolutionary force [59]. However, the underlying mechanism of this phenomenon is still debated and obscure. Faure et al. [60] considered that segregation distortion might due to the biological causes such as gametic and zygotic selection, non-homologous recombination, and the non-homologous or translocation loci on chromosomes. Zhang et al. [58] revealed that viability differences among genotypes and chromosome loss were possible reasons for segregation distortion. Others concluded that the segregation distortion could be due to environmental factors [56] or experimental errors [61]. In this study, 450 markers (37.85%) in the mapping population displayed significant distorted segregation (*P* < 0.05). The high segregation distortion ratio further indicated that genetic diversity between the parents of the mapped population is high [11]. Of 450 distorted markers, 421 markers showed clustered distribution in 54 SDRs, distributed between LGs. The clustering of distorted markers may due to the selection of gametophytes or sporophytes [62]. This phenomenon was widely reported in many plants [20, 35, 56]. Moreover, using distorted markers for linkage map construction could increase the genome coverage of the genetic map [35, 58], and may be beneficial for Quantitative trait locus (QTL) mapping [63, 64].

To our knowledge, the high-density genetic linkage map construction in this study is the first report for tree peony. This high-density linkage map will provide an important foundation for the QTL mapping, map-based gene cloning, and marker-assisted selection breeding for better understanding and improvement of tree peonies. In addition, 1115 SNPs (93.78%) on this map will be useful for comparative genomic studies [65] and association mapping [66], because they are sequence-tagged markers with co-dominant inheritance. More importantly, molecular markers on this high-density genetic map were developed at the whole genome level. So, this genetic map will also provide a significant platform for sequence scaffolds orientation and genome sequence assembly in tree peony [35, 67].

In conclusion, the present study demonstrates the utility of SLAF-seq technology for the large-scale identification of genetic markers in the tree peony, an organism without reference genomes. It also illustrates that the HighMap is an effective tool for high-density linkage map construction, using high-throughput sequencing data. Finally, we conducted the first high-density genetic linkage map for tree peony. We infer that the availability of such large-scale sequence markers, advanced high-throughput genotyping technology, and the high-density linkage map will serve as an important foundation not only for QTL mapping, map-based gene cloning, and molecular breeding, but also for orienting sequence scaffolds and assembling genome sequence for tree peonies in the future.

## Supporting Information

S1 Fig The first high density linkage map of tree peony. The linkage map of tree peony was an integrated map of *P*. *ostti* ‘FenDangBai’ and *P*. ×*suffruticosa* ‘HongQiao’, based on SLAF-seq, and was generated using HighMap. The name of the linkage is mentioned at the top of each LG. Distances of the loci (cM) are shown to the right and the names of loci are shown to the left of the linkage groups. Segregation distortion markers on the map are highlighted in red.
(TIF)

S1 Table Summary of Specific-locus Amplified Fragment Sequencing data in tree peony mapping population. (XLS)

S2 Table The sequences of 1189 SLAF markers which were mapped onto the genetic map of tree peony. (XLS)

S3 Table Details of genetic linkage map of tree peony constructed by HighMap and JoinMap4.1. (XLS)
