# Supplementary figures and images for: The First High-Density Genetic Map Construction in Tree Peony (Paeonia Sect. Moutan) using Genotyping by Specific-Locus Amplified Fragment Sequencing
Source: PLoS One. 2015 May 26;10(5):e0128584. doi: 10.1371/journal.pone.0128584 (PMC4444326; doi:10.1371/journal.pone.0128584)

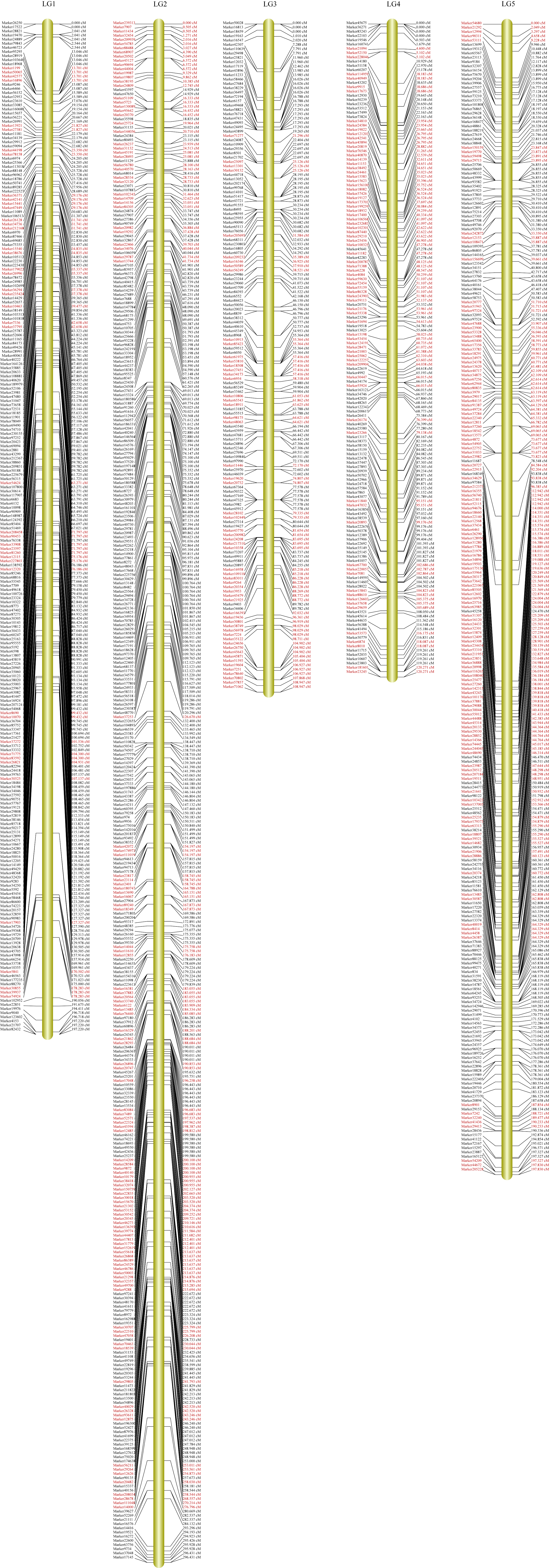

Supplement: S1 Fig — The linkage map of tree peony was an integrated map of P. ostti ‘FenDangBai’ and P. ×suffruticosa ‘HongQiao’, based on SLAF-seq, and was generated using HighMap. The name of the linkage is mentioned at the top of each LG. Distances of the loci (cM) are shown to the right and the names of loci are shown to the left of the linkage groups. Segregation distortion markers on the map are highlighted in red. (TIF) [file pone.0128584.s001.tif]
